# Who seeks child and adolescent mental health care in Kenya? A descriptive clinic profile at a tertiary referral facility

**DOI:** 10.1186/s13034-017-0151-x

**Published:** 2017-03-14

**Authors:** Judy Wanjiru Kamau, Olayinka O. Omigbodun, Tolulope Bella-Awusah, Babatunde Adedokun

**Affiliations:** 1 Department of Psychiatry, University of Nairobi, Nairobi, Kenya; 2 Department of Psychiatry, University of Ibadan, Ibadan, Nigeria; 3 Department of Public Health, University of Ibadan, Ibadan, Nigeria

**Keywords:** Child mental health services, Child psychiatry, Clinic profile, Comorbidity, Substance use, Depression

## Abstract

**Background:**

The presence of psychiatric morbidity in the child and adolescent age group is demonstrable in various studies conducted in various settings in Kenya. This study set out to determine the psychiatric morbidity and socio-demographic profile of patients who eventually present for care at a tertiary specialist child and adolescent mental health clinic in Kenya. Knowledge of the patterns of presentation of disorders is crucial for planning of service scale up as well as serving as a useful training guide.

**Methods:**

This was a cross sectional descriptive study of 166 patients and their guardians presenting to the child and adolescent mental health clinics at a tertiary referral hospital in Nairobi, Kenya. Data was collected using a researcher designed socio-demographic questionnaire and the Kiddie-schedule for affective disorders and schizophrenia-present and lifetime (KSADS-PL 2009 Working Draft) and analysed using Statistical Package for Social Scientists.

**Results:**

There were more males (56%) than females in this study and the participant’s mean age was 13.6 years. Substance abuse disorders were the most prevalent presentation (30.1%) followed by depressive disorders (13.9%), with most referrals to the clinic coming from medical practitioners and teachers. The mean time to accessing care at the clinic after the onset of symptoms was 16.6 months, with the longest time taken to specialist care being 183 months.

**Conclusions:**

The findings from this study will go a long way to support the establishment of programs that improve timely child and adolescent mental health service delivery. The involvement of various stakeholders such as the education sector and the community is key in the development of these programs.

## Background

Kenya, a low middle-income country in Africa with a population of 44 million, has a largely youthful population comprised of 48% children and adolescents [1].

The existence of psychiatric morbidity in children and adolescents living in Kenya has been documented in several prevalence studies from various settings. A pilot study by Kangethe [2] found a psychiatric morbidity prevalence rate of 20% among children and adolescents aged 5–15 years attending a primary health care facility. Mulupi [3] found that 41.2% of 255 adolescents had psychiatric disorders in a similar setting. A comparative study of psychiatric morbidity among rural and urban primary school pupils revealed a 26% psychiatric morbidity rate in the rural students compared to a 41.2% rate in their urban counterparts [4].

Mental health care needs are also demonstrated in other cohorts of children living in Kenya. This includes children infected with Human Immunodeficiency Virus (HIV), who have in some settings a psychiatric morbidity prevalence of 48.8%, and in sexually abused children where there is a prevalence of 61% as well as in young criminal offenders with a prevalence of 44.4% [5–7].

Although the number of psychiatrists has increased over the years, so has the Kenyan population, giving an estimated ratio of one psychiatrist to a population of about half a million people with most psychiatrists practicing in the capital, Nairobi [8, 9]. These are extremely poor ratios compared to those found in high income countries [10]. Psychiatry services in primary health care may be provided by clinical officers and nurses, and where available medical officers. There is a dearth of child and adolescent mental health specialists in the country and at the time of the study, there was only one in clinical practice and only two specialist child and adolescent mental health (CAMH) clinics that catering specifically to the needs of children in Kenya.

There is a need to build up child mental health services for the child and adolescent population in the country and a baseline knowledge of the profile of those who seek care would greatly contribute to the determination target areas during the scale up of these services.

The aim of this study was to define the profile of patients who eventually sought specialist child and adolescent mental health services in Kenya in terms of the pattern of psychiatric morbidity as well as the socio-demographic profiles, referral source and time taken to get to the specialist CAMH clinics after the onset of symptoms. The findings of the study would help to guide the development of additional services and capacity building for CAMH services.

## Methods

This was a cross sectional descriptive study that targeted 166 children and adolescents aged 0–18 years and their caregivers attending the child and adolescent mental health clinics at the Kenyatta National Hospital, the largest tertiary referral hospital in Kenya, located in the capital Nairobi. The data was collected as part of a study on pathways to child and adolescent mental health services in Kenya, looking at factors influencing help-seeking in terms of choice of type of care, psychiatric morbidity, sources of referral along the way and time to seek help after onset of symptoms. The sample size was calculated using the Cochran formula for descriptive studies, with the desired level of precision set at 5% [11]. As this was a pathways to care study, the hypothesized prevalence level was set at 72% (proportion of patients receiving care from medical facilities as a first point of care) from previous studies [12]. This was then adjusted to the Kenyatta National Hospital clinic population at the time of the study. Ethical approval to conduct the study was obtained beforehand from the Kenyatta National Hospital/University of Nairobi Ethics and Research Committee. Participants were only included in the study if they were seeking care at the Kenyatta National Hospital child and adolescent mental health clinics (both new patients and those already on clinic follow-up), were aged between 0 and 18 years, if the guardians gave informed written consent and the children and adolescents gave assent to the study. Purposive sampling technique was used to collect data until the desired sample size was reached. No refusals were encountered during the study.

A researcher designed socio-demographic questionnaire, was used to collect information on sex, age, referral source to the clinic, and religion among others. The Kiddie-schedule for affective disorders and schizophrenia-present and lifetime (KSADS-PL 2009 Working Draft), a semi structured tool was used for diagnostic purposes. Its usage is freely permitted for research and clinical usage by non-profit organisations. It is designed for children and adolescents aged 6–18 years to assess current and past episodes of psychiatric morbidity according to diagnostic and statistical manual of mental disorders-4th edition (DSM IV) criteria and has a section for assessment of suicide risk. It covers most diagnoses in DSM IV in children except Intellectual disabilities and Somatoform disorders (these were diagnosed clinically). The tool has not been used previously on the Kenyan population but has been used in other African countries wholly or in part as a tool or as a gold standard to validate other screening instruments [13, 14]. It was used in tandem with the Children’s Global Assessment Scale to assess impairment of function on a continuous scale of 0–100 for children aged 4–18 years.

One researcher collected data over a 12-week period. The administration of the questionnaires by the researcher took an average of between 30 min and an hour and a half. All tools were directed to the parent/guardian but the K-SADS was also administered to the child for a corroborative history.

Collected data was statistically analysed using S.P.S.S (Statistical Package for Social Scientists) software version 20 [15]. Personal and family socio demographic variables of the study participants were analysed and presented in their various frequencies and the means of continuous variables acquired. Clinical variables were tabulated in order of frequency of occurrence and comparisons were made for age and gender. Means, mode and median of the duration of time taken to present for care at the tertiary care facility (study site) were also acquired.

## Results

### Socio demographic characteristics

One hundred and sixty-six participants were enrolled into the study. The ages of the children and adolescents ranged from 2 to18 years with a mean of 13.6 ± 4.16 years. Ninety-three (56%) were male, with a female: male ratio of 1:1.2. Out of the 32 children below 10 years, 25 of them (78.1%) were male while 7 (21.9%) were female. Among the 134 adolescents in the sample, 68 (50.7%) were male and 66 (49.3%) were female. This difference in gender across the ages at presentation was found to be statistically significant (p = 0.005). Seventeen (10.2%) of the children and adolescents were not in school for various reasons. Two (1.2%) had dropped out of school, 5 (3%) had been expelled from school, 5 (3%) were not in school due to the mental disability and 5 (3%) were not yet of school age. The personal socio-demographic characteristics of the study participants are displayed in Table 1.

**Table 1:** Personal socio demographic characteristics of the study participants (N = 166)

Variables	n (%)
Age (years)	
0–4	8 (4.8)
5–9	24 (14.5)
10–14	37 (22.3)
15–18^b^	97 (58.4)
Total	166 (100)
Gender	
Male	93 (56.0)
Female	73 (44.0)
Total	166 (100)
School status/gradeª	
Pre primary	12 (7.2)
Primary	35 (21)
Secondary	89 (53.6)
Post-secondary	4 (2.4)
Special school	9 (5.4)
Not in school	17 (10.2)
Total	166 (100)

^a^Pre-primary (4–6 years of age) Primary [8 years of schooling (class 1–class 8)] Secondary [four years of schooling (form 1–form 4)]

^b^Range of ages restricted by scope of survey (under 1–18 years)

Forty-six (27.7%) of the participants were from single parent households, while 9 (5.4%) were double orphans. Of the non-parent guardians, 16 (9.6%) of them were blood relatives. The family characteristics of the study participants are displayed in Table 2.

**Table 2:** Family characteristics of the study participants

Variables	n (%)
Primary guardian	
Biological parent	147 (88.6)
Blood relative	16 (9.6)
Non relative	3 (1.8)
Total	166 (100)
Parental status	
Married	111 (66.9)
Separated or divorced	37 (22.3)
Maternal orphan	1 (0.6)
Paternal orphan	8 (4.8)
Both parents dead	9 (5.4)
Total	166 (100)
Educational status of father	
No formal education	3 (2.7)
Primary school	13 (11.6)
Secondary school	43 (38.4)
Tertiary	54 (47.3)
Total	112 (100)
Occupational status of fatherª	
Professional	41 (36.60
Non-professional	69 (61.6)
No employment	2 (1.8)
Total	112 (100)
Educational status of mother	
No formal education	3 (1.9)
Primary school	30 (19.2)
Secondary school	70 (44.9)
Tertiary education	53 (34.0)
Total	156 (100)
Occupation status of motherª	
Professional	32 (20.5)
Non-professional	96 (61.5)
No employment/homemaker	28 (18.0)
Total	156 (100)
Geographical area (province)	
Eastern	9 (5.4)
Central	39 (23.5)
Rift valley	10 (6.0)
Nyanza	4 (2.4)
Nairobi (site of study)	104 (62.7)
Total	166 (100)
Religion	
Christianity	165 (99.4)
Islam	1 (0.6)
Total	166 (100)

^a^Professional: requires tertiary education; Non-professional: requires little or no formal education

**Fig. 1:**
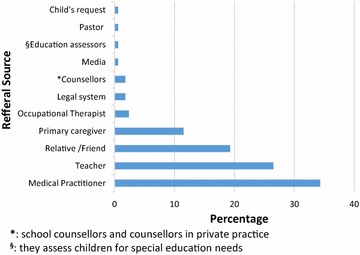
Sources of referral to the CAMH clinic

Medical practitioners referred 57 (34.3%) of the study participants to the CAMH clinics, teachers referred 44 (26.5%), while 19 (11.5) of the participants were directly brought in by the primary caregiver. One participant (0.6%) came into the clinic after getting information about the clinic from the media. The referral source information is displayed in Fig. 1.

### Time between onset of symptoms and getting to the child and adolescent mental health clinic

The longest time taken between onset of symptoms and finally reaching the child and adolescent mental health clinics was 183 months (15.25 years). The mean time taken was 16.6 months (SD: 26.03), while the inter quartile range was 22.6 months. Most of the caregivers took 1–6 months to get to the mental health clinic after onset of symptoms, while 12 (7.2%) took more than four years to get to the CAMH clinic as further illustrated in Table 3.

**Table 3:** Time between onset of symptoms and getting to the CAMH clinic (N = 166)

Time between onset of symptoms and care at CAMH clinic	n (%)
Within a week	29 (17.5)
More than a week but less than a month	7 (4.2)
1–6 months	50 (30.1)
7–12 months	22 (13.2)
13–24 months	24 (14.5)
25–36 months	14 (8.4)
37–48 months	8 (4.8)
49 months/more	12 (7.2)

### Mental and physical disorders in the study sample

Table 4 displays the clinical characteristics of the study sample. Substance use disorders related to cannabis use were the most common psychiatry diagnosis followed by major depression. Intellectual disability was diagnosed in 17 (10.2%) of the children and adolescents while seizure disorders 18 (10.8%) were the most common of the physical conditions. Other physical conditions found in the sample were cerebral palsy 1 (0.6%), HIV 1 (0.6%), headache 1 (0.6%) and hearing difficulties 2 (1.2%). Twenty-three (13.7%) of the children and adolescents in the study reported experiencing suicidal ideation, and 7 (4.2%) of them reported having attempted suicide at least once.

**Table 4:** Frequency distribution of mental and physical disorders in the study sample (N = 166)

Disorders	Number	%
Psychotic disorders and bipolar disorders		
Schizophrenia	9	5.4
Schizoaffective disorder	1	0.6
Schizophreniform disorder	3	1.8
Brief psychotic disorder	1	0.6
Bipolar disorder	7	4.2
Depression, anxiety and related disorders		
Major depression and dysthymia	23	13.9
Anxiety disorders	11	6.6
Somatoform disorders	10	6.0
Adjustment disorders	6	3.6
Disruptive disorders		
Attention deficit hyperactivity disorder (ADHD)	20	12.1
Conduct disorder	12	7.2
Oppositional defiant disorder	9	5.4
Disruptive disorder not otherwise specified	2	1.2
Substance related disorders		
Tobacco use	10	6.0
Alcohol use (abuse and dependence)	12	7.2
Cannabis use (abuse and dependence)	24	14.5
Stimulant abuse	3	1.8
Cocaine dependence	1	0.6
Autism spectrum disorders	21	12.7
Physical disorders		
Seizure disorder	18	10.8
HIV	1	0.6
Cerebral palsy	1	0.6
Others (headache and hearing)	3	1.2
Suicidality	23	13.9
Intellectual disability	18	10.8
Others		
Tic disorders	2	1.2
Enuresis	3	1.8
Other conditions that may be a focus of clinical attention (related to social environment, social support and school problems)	18	10.8

N.B. Due to presence of comorbidities, the total n (%) will be more than 100%

**Fig. 2:**
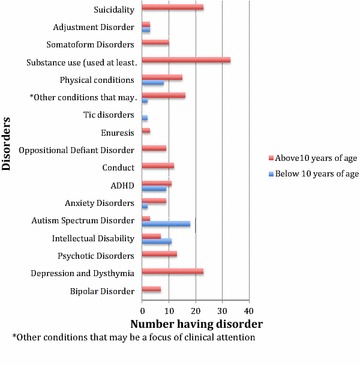
Comparison graph of disorders in participants below 10 years versus those above 10 years

Figure 2 displays the prevalence of the disorders by age group, comparing those below 10 years to those above 10 years. Autism spectrum disorders were highest in the lower age group. There were no substance use disorders in the lower age group.

**Fig. 3:**
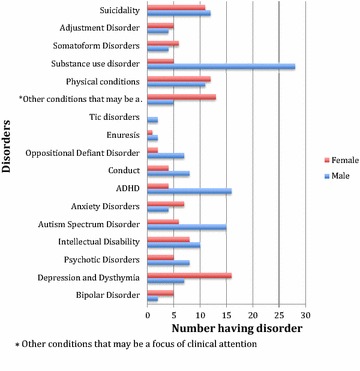
Comparison graph of disorders presenting in males versus females

More female participants had depression (n = 16) compared to the male participants (n = 7) while 28 males had a substance abuse problem compared to females (n = 5). This is displayed on Fig. 3.

Out of the 166 children and adolescents enrolled in the study, 69 (41.6%) met the diagnostic criteria for more than one disorder and 16 (9.6%) of the study participants had used more than one substance of abuse.

Twelve out of the 23 (52.1%) children and adolescents who had suicidality were diagnosed to have depression. Depression had the highest rate of associated comorbidities followed by substance use disorders.

The Children’s Global Assessment Scale was used to assess 162 children aged above 4 years. More than half of them 89 (54.9%) were grouped into the severe impairment of function category, while the normal and mild impairment prevalence was 32 (17.8%) and 41 (25.3%) respectively.

## Discussion

The study participants were aged 2–18 years with a mean age of 13.6 years (SD = 4.16), with the males predominating the sample in all age groups. The gender characteristics observed in this study are similar to those reported in other child clinic populations [16–18]. A similar study of 127 children and adolescents referred to a tertiary care facility in Ibadan, Southwest Nigeria, had a mean age of 12.7 years and a higher proportion of males (62%) presenting at the child and adolescent mental health clinic [16].

Compared to the lower age groups (<10 years) the proportion of females using the mental health service in this study was noted to increase in the adolescent age group. This observation is similar to findings in child psychiatry clinic populations in South Africa and the United States of America [19, 20]. Epidemiological studies reveal a preponderance of depression in females around the age of 13 years resulting in the increased proportion of girls in mental health facilities [21], a finding that was consistent with the current study. In the youngest age group, the reasons for the much larger proportion of males observed can be accounted for by a well established fact that all developmental disorders are commonly found in boys [22].

Most of the study participants who came to the child and adolescent mental health service at the Kenyatta National Hospital, Nairobi were from Nairobi and its environs within an 80 km radius. The fewest came from Nyanza province, which is furthest from the Kenyatta National Hospital. The presence of Moi Teaching and Referral Hospital (Kenya’s second national referral hospital after Kenyatta National Hospital) 313 km North west of Nairobi and whose immediate catchment area covers Rift valley, Western and Nyanza provinces would explain why a low proportion of participants came from this distant area. There were no participants from the Coast province, which is more than 400 km from Nairobi. This region however has mental health services manned by psychiatrists, which the community is able to access. There were no participants from North Eastern province, which has no psychiatrists and is far from both Kenyatta National Hospital and Moi Teaching and Referral Hospital (367 and 650 km respectively). This region of Kenya is fraught with conflict and instability as a result of terrorist attacks and may account for the lack of psychiatrists and mental health services in a region with much need.

There was only one Muslim among the study participants while the rest were Christians, probably because the predominant religion in Kenya is Christianity (82%), and the few Muslims predominantly live in the Coastal and North Eastern provinces, far from the site of the study [23].

Individuals suffering from psychiatric disorders are more likely to have a truncated education due to the disability from the disorder [24]. More than 90% of the participants of school going age in the study were attending either mainstream or special school. This proportion of in-school attendees at the child and adolescent mental health service is much higher when compared to findings from similar studies in West Africa. Omigbodun [16] reported that over a quarter (27.6%) of children who presented in a child and adolescent mental health clinic at a tertiary health facility in Ibadan, Southwest of Nigeria were not attending school. Similarly, a study of children presenting to a tertiary health facility study in Maiduguri, Northeast of Nigeria revealed that over half of their study participants with a mean age of 12.3 years were not in school [25]. A reason for this difference may be that service users presenting to these tertiary facilities in both North and South of Nigeria had more severe and disabling disorders than in the Kenyan clinical setting. A recent report from United Nations Children’s Fund revealed that the West African region had the lowest primary school enrolment rates in the world and this could have contributed to the higher proportion of participants not in school in the Nigerian clinical setting [26].

Pedrini et al. [27] in an Italian study of 399 patients seeking CAMH services for the first time found teachers as the main sources of referral (36%) closely followed by doctors (32%). In Nepal however, the main referral source for the 539 study participants attending a child guidance clinic at a tertiary level teaching hospital was primarily by medical personnel from the hospital itself and other hospitals. Twenty percent of the participants were self-referred while 15.6% of them were referred by other sources such as friends, neighbours, relatives, other patients and traditional healers as well as through information from the media. Teachers did not appear as sources of referral [28]. In the current study, medical practitioners (34%) were the main sources of referral for care at the CAMH clinics, closely followed by teachers (26.5%) then friends and relatives (19%). As this study was conducted at a tertiary referral facility, it would be expected that medical personnel would be involved in the referral process to the CAMH clinics. Teachers are highlighted as key individuals for the recognition and appropriate referral for care. Social networks also seem to play a role in the overall care of children and adolescents as demonstrated by the role played by friends and relatives in the referral process in the current study.

A 5 year survey of children and adolescents referred to a neuropsychiatry hospital in Lagos, Southwest Nigeria revealed that psychotic disorders (38%), were most prevalent disorders in that clinical setting closely followed by seizure disorders (34%) but very low depressive disorder rates (1.3%) [29]. Similarly, a study conducted at a child and adolescent mental health clinic also in the Southwest region of Nigeria revealed high rates of psychotic disorders (32.3%), low depressive disorder rates (1.6%) and seizure disorders at 11% [16]. Conversely, Raman and van Rensburg [19] found ADHD (24%) and depressive (17.8%) disorders were the most prevalent diagnosis in an urban child mental health clinic at a tertiary children’s hospital in Johannesburg, South Africa. A study featuring 100 participants aged between 13 and 18 years attending a psychiatry clinic at a tertiary care hospital in Nepal found depressive disorders were most prevalent at 20%, followed by anxiety disorders (16%) [30]. In the current study, mental disorders most diagnosed were substance use related disorders, specifically cannabis use followed by depressive disorders. Cultural contexts and beliefs may be associated with parental differences in tolerance thresholds and their perceptions on whether or not a problem behaviour is mental health related [31].

The high presentation of substance use disorders to the child and adolescent mental health clinic in the Kenyan setting could be due to the intensified public and media campaign against drug and alcohol abuse in the country by the National Authority for the Campaign Against Alcohol and Drug Abuse [32].

The current study also had a high comorbidity rate of 41.6%, with most comorbidities relating to major depression and substance use disorders, and there was also presence of physical disorders. This is consistent with other studies [27–29]. With comorbidity comes associated increased mortality and functional impairment. Collaborative care approaches with medical and mental health linkages in service delivery would prevent fragmentation of services to the patient and improve the provision of holistic quality of care to the patients [33].

## Limitations

This was a clinical profile conducted at a tertiary referral facility in Kenya. This would not be wholly representative of the Kenyan population as more than 50% of the catchment area of this facility was the capital city and the environs, and would not be inclusive of the rural population. Although a trained CAMH clinician collected the clinical data, the tool used (K-SADS) has not been validated in the Kenyan population. A non-randomised sampling technique was applied in this study and would have introduced bias.

## Conclusions

While the presentation of mental health problems in the child and adolescent population is similar in some aspects to other centres in Africa and other continents, substance use disorders presentation in this study was much higher both singularly or as a comorbidity. Teachers were also seen to play a significant role as referral agents to the specialist mental health clinics. The data in this study will be very useful in the support of programs that improve CAMH service delivery such as school based programs targeting substance use and other mental health disorders in this population as well as creating awareness to the community and physicians on the existence of services to facilitate timely referrals and markedly reduce the time to eventual specialist care.

## Abbreviations

CAMH: child and adolescent mental health
DSM-IV: diagnostic and statistical manual of mental disorders (fourth edition)
K-SADS: Kiddie-schedule for affective disorders and schizophrenia
SD: standard deviation
